# Phase I Clinical Trial: Evaluating the Efficacy, Safety, and Patient Satisfaction of Platelet‐Rich Plasma (PRP) Injections and Microneedling for Androgenetic Alopecia Treatment

**DOI:** 10.1111/jocd.70408

**Published:** 2025-09-17

**Authors:** Mohammad Ali Nilforoushzadeh, Masoumeh Roohaninasab, Elham Behrangi, Alireza Jafarzadeh, Maryam Nouri, Solmaz Zare, Sona Zare

**Affiliations:** ^1^ Skin Repair Research Center Jordan Dermatology and Hair Transplantation Center Tehran Iran; ^2^ Skin and Stem Cell Research Center Tehran University of Medical Sciences Tehran Iran; ^3^ Department of Dermatology, Hazrat Fatemeh Hospital, School of Medicine Iran University of Medical Sciences Tehran Iran; ^4^ Laser Application in Medical Sciences Research Center Shahid Beheshti University of Medical Sciences Tehran Iran; ^5^ Pars Fundamental Bio Structure Company Tehran Iran; ^6^ Stem Cell and Regenerative Medicine Institute Sharif University of Technology Tehran Iran; ^7^ Department of Mechanical Engineering Sharif University of Technology Tehran Iran

**Keywords:** androgenetic alopecia, injection, microneedling, platelet‐rich plasma

## Abstract

**Introduction:**

Platelet‐rich plasma (PRP) has been widely utilized in dermatological treatments, leading to the development of various administration methods. This study aims to evaluate and compare the effectiveness of PRP injections and microneedling techniques for treating androgenetic alopecia (AGA) in clinical practice.

**Materials and Methods:**

A total of 40 participants diagnosed with AGA, aged between 18 and 50 years, were randomly assigned to receive either PRP injections or microneedling treatments. Each patient underwent two treatment sessions, with a 1‐month interval between them. Hair density and thickness measurements were performed using TrichoScan before treatment and 2 months after the final session. Statistical analysis was conducted using SPSS software.

**Results:**

Among the participants, 28.57% were male and 71.43% were female, with an average age of 40.2 years (±7.7). In the PRP injection group, hair count increased by approximately 62.4% (from 17.40 ± 2.074 to 28.26 ± 6.229), and hair thickness improved by 58.6% (from 58.0 ± 25.01 to 92.0 ± 16.31). In the microneedling group, hair count increased by 88.4% (from 14.71 ± 3.988 to 27.71 ± 4.499), while hair thickness improved by 51.3% (from 49.0 ± 11.30 to 74.14 ± 22.42). Although both groups showed statistically significant improvements (*p* < 0.05), the intergroup differences in hair count and thickness were not statistically significant (*p* > 0.05).

**Conclusion:**

The findings of this study confirm PRP as a viable treatment option for AGA. Both injection and microneedling methods provided therapeutic benefits. Additionally, microneedling appeared to enhance patient satisfaction and treatment tolerance.

## 1 Introduction

Androgenetic alopecia (AGA) is a prevalent hair loss condition affecting individuals worldwide [1, 2]. It manifests through a gradual decline in scalp hair density, displaying characteristic male and female patterns, along with disruptions in the hair growth cycle and follicular shrinkage [3, 4]. Various factors, including genetic predisposition, hormonal imbalances, environmental aspects, and psychological influences, contribute to the onset and progression of AGA [5, 6].

Both male and female pattern baldness become more common with age, impacting over 50% of people above 50 years old [3, 4, 5]. While hereditary and hormonal influences are the main causes of AGA in men, the condition in women is more multifaceted, involving numerous contributing factors. Hair loss in women can significantly affect their physical appearance, emotional health, and self‐esteem, as societal norms tend to be less accepting of female baldness [2, 7, 8]. Studies indicate that AGA is more commonly observed in Caucasians, with variations in prevalence based on age and ethnicity [5].

Several treatment methods have been explored for managing AGA, including medications, hair transplants, and physical therapies such as laser procedures and growth factor‐based interventions [9, 10]. However, the U.S. Food and Drug Administration (FDA) has approved only two pharmacological treatments for hereditary and hormonal hair loss: minoxidil and finasteride [3]. These medications require continuous, long‐term use, and hair regrowth often diminishes once treatment is discontinued [11]. Moreover, nonsurgical therapies have demonstrated limited success in managing AGA [8, 12]. Consequently, AGA remains a complex issue, requiring further research into more effective treatment alternatives [8].

Given the widespread occurrence of hereditary and hormonal hair loss, its effect on an individual's self‐perception, the slow hair regrowth associated with current treatments, their necessity for prolonged use, and their limited effectiveness with potential side effects, discovering a more efficient and practical therapy is essential [1, 13, 14]. AGA shares characteristics with tissue damage and repair processes, where growth factors stimulate cellular activity and attract necessary components for healing. This has led to the hypothesis that regenerative approaches, such as platelet‐rich plasma (PRP) therapy, could support hair follicle repair [11].

PRP is a component derived from plasma that contains an increased concentration of platelets, providing a rich source of growth factors such as transforming growth factor‐β (TGF‐β), vascular endothelial growth factor (VEGF), platelet‐derived growth factor (PDGF), insulin‐like growth factor (IGF), and epidermal growth factor (EGF) [11]. PRP has gained significant traction in medical, surgical, wound healing, and cosmetic applications [15, 16, 17]. More recently, it has emerged as a promising, minimally invasive option for promoting hair regrowth in AGA patients [10, 18]. PRP can be delivered via direct scalp injections or through microneedling [19]. The microneedling technique involves creating tiny skin injuries that enhance PRP absorption, leading to improved treatment outcomes; though some adverse effects may occur [20, 21].

This study seeks to assess and compare the effectiveness of PRP administration through injection and microneedling in patients with AGA.

## 2 Materials and Methods

### 2.1 Study Design and Participants

This study involved 40 patients, aged 18–50, presenting with AGA at a dermatology clinic. Ethical approval was granted by the Research Ethics Committee (IR.TUMS.MEDICINE.REC.1400.031), and the study was officially registered (IRCT20200127046282N6).

Before enrollment, all participants were thoroughly briefed on the study's aims, procedures, possible benefits, risks, and follow‐up requirements. Each individual provided written informed consent. Participants were randomly allocated into two groups: one receiving PRP injections at hair loss sites (*n* = 20) and the other undergoing PRP application via microneedling (*n* = 20). Both groups received two treatment sessions, spaced 1 month apart, with follow‐up assessments conducted 2 months posttreatment. Additionally, all patients were prescribed standard AGA therapy—1 mg of finasteride daily for men and cyproterone compound tablets for women (administered from day 5 to day 21 of their menstrual cycle).

### 2.2 Inclusion and Exclusion Criteria

#### 2.2.1 Inclusion Criteria

- Diagnosis of AGA
- Age between 18 and 50 years
- Willingness to participate with informed consent
- Hamilton scale scores of 2–5 for men and Ludwig scale scores of 1–3 for women

#### 2.2.2 Exclusion Criteria

- Platelet dysfunction or low platelet count
- Use of anticoagulant medications
- Presence of malignancies
- Women with hyperprolactinemia or hormonal imbalances
- Active infections or wounds at the treatment site

### 2.3 PRP Preparation and Administration

PRP was prepared following the American Association of Blood Banks' guidelines using a two‐step centrifugation process. Whole blood (10 mL) was collected in sodium citrate tubes and centrifuged using a 320 Universal device (Hettich, Germany). The initial centrifugation (160 g, 10 min) separated PRP from whole blood; followed by a second centrifugation (400 g, 10 min) to isolate platelet‐poor plasma. The platelet‐rich fraction was extracted using a sterile kit from Persian Bio‐Based Production (PBBP) Company.

A total of 2 cc of PRP was injected into androgen‐related scalp areas using a 30G needle.

### 2.4 Microneedling Procedure

A dermaroller with 1.5 mm needles was used to perform microneedling across the affected scalp regions in vertical, horizontal, and crosswise motions until pinpoint bleeding appeared. PRP was then applied topically to the treated areas. Each procedure lasted approximately 10–20 min per patient.

### 2.5 Assessment Methods

Evaluations were conducted at baseline and 2 months after the final treatment using the TricoScan device to measure hair density (*n*/cm^2^) and thickness. A designated scalp region was analyzed for quantitative assessment.

#### 2.5.1 Pull Test

A standardized hair pull test was conducted by the same physician at each visit. Between 20 and 60 hairs were gently pulled near the scalp; shedding of more than 10% was considered a positive test result.

#### 2.5.2 Patient Global Assessment Score

Participants rated their satisfaction using a four‐point scale:

- 0 = Poor
- 1 = Acceptable
- 2 = Good
- 3 = Excellent

### 2.6 Statistical Analysis

Quantitative variables, such as age, hair density, and hair thickness, were expressed as mean ± standard deviation, while categorical data were presented as percentages. Due to the limited sample size, the Wilcoxon nonparametric test was used to compare pre‐ and posttreatment hair density and thickness within each group. The Mann–Whitney test was applied to determine differences in changes between the two treatment groups. Statistical analysis was performed using SPSS software (Version 24), with significance set at *p* < 0.05.

No artificial intelligence–generated content (AIGC) tools, including large language models such as ChatGPT or similar, were used in the development, writing, or analysis of this manuscript. All content was prepared solely by the authors.

## 3 Results

### 3.1 Participant Demographics

Among the study participants, 28.57% were male, while 71.43% were female. The mean age of the group was 40.2 ± 7.7 years.

### 3.2 PRP Injection Outcomes

**FIGURE 1:**
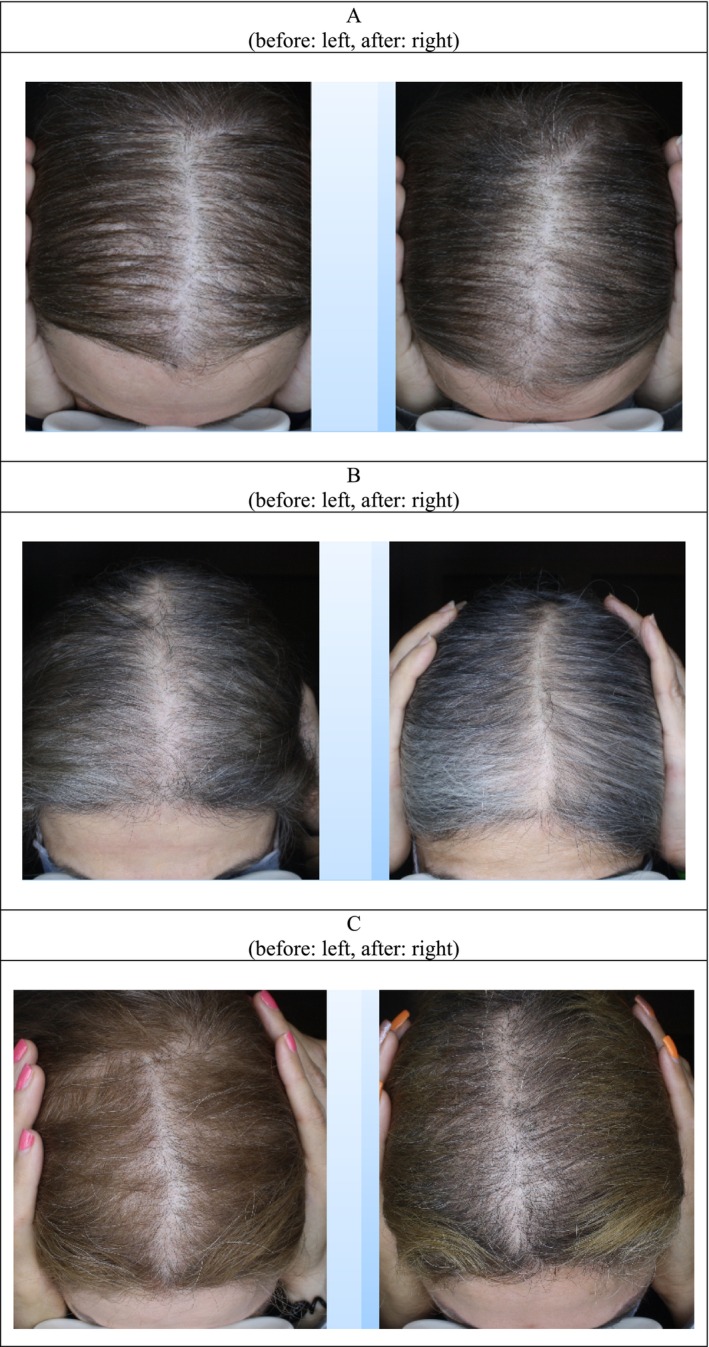
Image of before and 2 months after treatment by PRP injection in three patients' (A–C).

**FIGURE 2:**
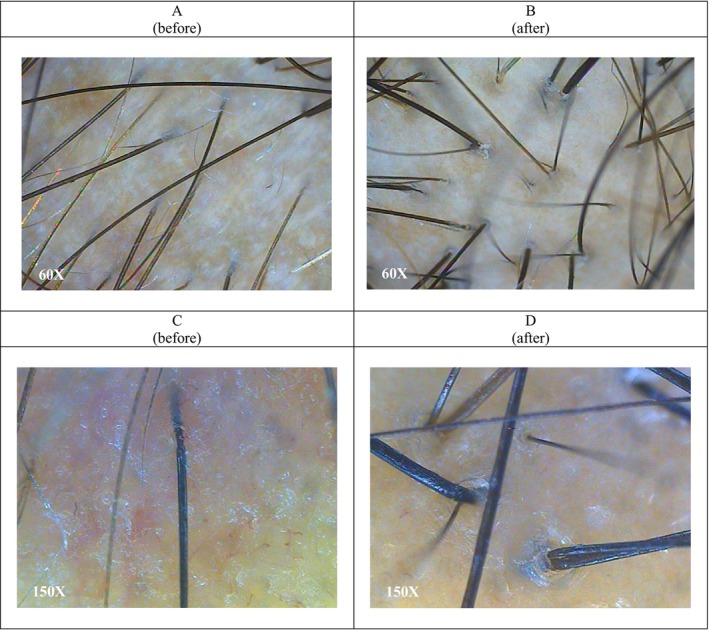
Image of before (A, C) and 2 months after (B, D) of treatment by PRP injection (60× and 150× lens).

Hair count increased significantly from a baseline of 17.40 ± 2.074 to 28.26 ± 6.229 after PRP injection (*p* = 0.043). Similarly, hair thickness showed a statistically significant improvement, rising from 58.0 ± 25.01 to 92.0 ± 16.31 posttreatment (*p* = 0.043) (See Table 1, Figures 1 and 2).

**TABLE 1:** Comparison of measured parameters at baseline and after treatment in PRP injection group.

Variables	Time	Mean ± SD	Percentiles			*p*
			25	50 (median)	75	
*n* = 5						
(Triple ×60) hair number in 4*5 Mm	Before	17.40 ± 2.074	15.5	17.0	19.5	0.043
	After	28.6 ± 6.229	24.0	27.0	34.0	
(Kpl ×150) hair thickness in Mm	Before	58.0 ± 25.01	36.0	61.0	78.5	0.043
	After	92.0 ± 16.31	78.5	87.0	108.0	

### 3.3 PRP Microneedling Outcomes

For patients undergoing PRP with microneedling, hair count rose from 14.71 ± 3.988 before treatment to 27.71 ± 4.499 after treatment, demonstrating a significant increase (*p* = 0.018). Hair thickness also improved from 49.0 ± 11.30 to 74.14 ± 22.42 (*p* = 0.018) (See Table 2).

**TABLE 2:** Comparison of measured parameters at baseline and after treatment in PRP microneedling group.

Variables	Time	Mean ± SD	Percentiles			*p*
			25	50 (median)	75	
*n* = 7						
(Triple ×60) hair number in 4 × 5 Mm	Before	14.71 ± 3.988	12.0	13.0	18.0	0.018
	After	27.71 ± 4.499	25.0	30.0	32.0	
(Kpl ×150) hair thickness in Mm	Before	49.0 ± 11.30	37.0	53.0	58.0	0.018
	After	74.14 ± 22.42	47.0	84.0	92.0	

### 3.4 Comparison of PRP Injection vs. Microneedling

Tables 1 and 2 present hair density and thickness changes for both treatment groups, confirming significant gains in both variables.

**FIGURE 3:**
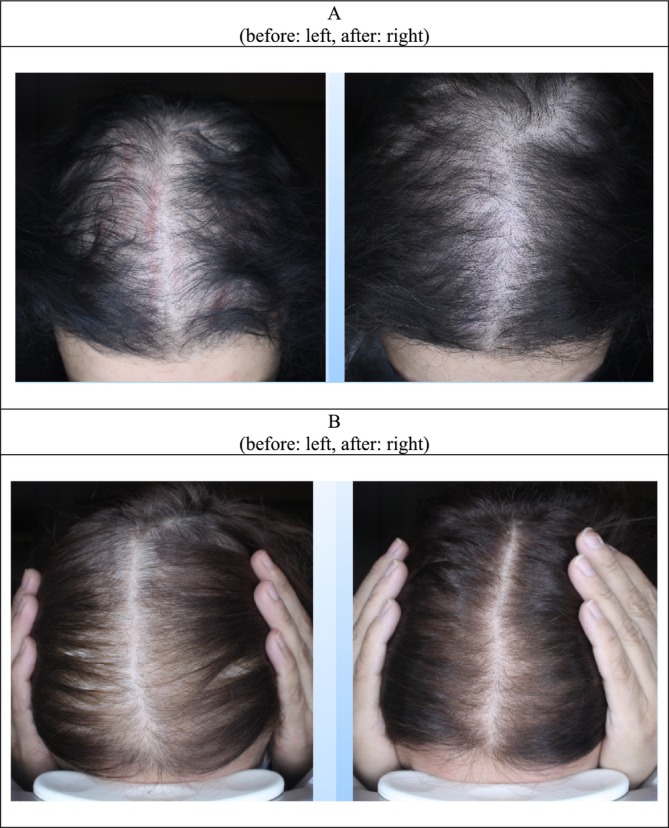
Image of before and 2 months after treatment by PRP microneedling in three patients' (A, B).

**FIGURE 4:**
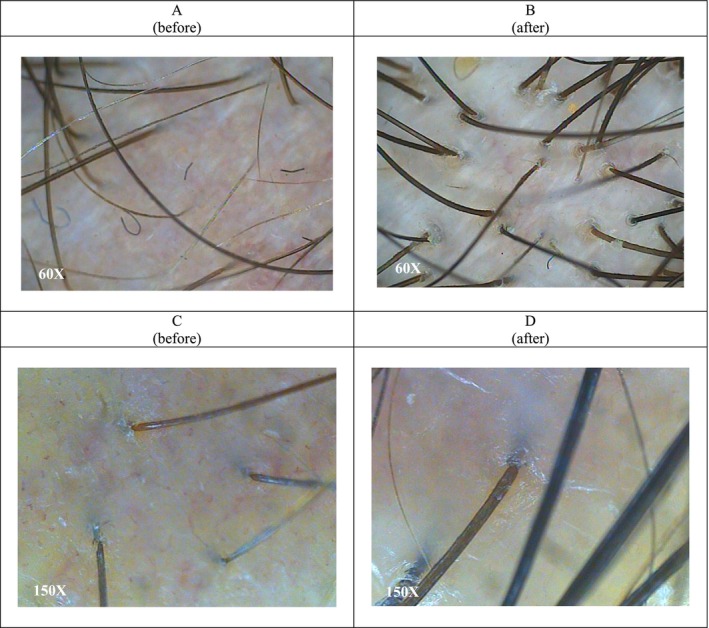
Image of before (A, C) and 2 months after (B, D) of treatment by PRP microneedling (60× and 150× lens).

- Hair count improvements were more prominent in the microneedling group; though this difference was not statistically significant (*p* = 0.219).
- Hair thickness increased slightly more in the PRP injection group, but the difference compared to microneedling was not statistically significant (*p* = 0.570) (See Table 3, Figures 3 and 4).

**TABLE 3:** Comparison of the rate of parameter changes in PRP injection and microneedling groups.

Variables	Group	Mean ± SD	Percentiles			*p*
			25	50 (median)	75	
(Triple ×60) hair number in 4 × 5 Mm	PRP	64.69 ± 30.29	35.15	66.67	93.26	0.219
	PRP + Micro	95.18 ± 39.84	66.67	88.24	150.0	
(Kpl ×150) hair thickness in Mm	PRP	76.64 ± 59.60	29.35	42.62	140.95	0.570
	PRP + Micro	51.80 ± 39.73	23.68	50.79	69.811	

### 3.5 Patient Satisfaction and Pain Perception

Treatment satisfaction was higher among microneedling patients, with 69% rating their results as “excellent” (score 3), compared to 58% in the PRP injection group.

Pain tolerance was also higher in the microneedling group, with 63% reporting good pain tolerance, while 49% in the PRP injection group expressed similar comfort levels. Although microneedling appeared to cause slightly less discomfort than direct PRP injections, the difference was not statistically significant.

## 4 Discussion

Although pharmaceutical treatments for AGA are available, their long‐term use and potential side effects make them less appealing to many patients [11]. As a result, PRP has emerged as an alternative therapeutic option, showing promise not only for AGA but also for various dermatological conditions [15, 22, 23]. Different PRP delivery techniques have been explored, with studies reporting varying levels of effectiveness [10].

This study compared PRP injection and microneedling methods for treating AGA to determine their relative effectiveness. Both approaches significantly enhanced hair count and thickness, but no statistically significant difference was observed between them. Hair count increased by an average of 64.69 in the PRP injection group and 95.18 in the microneedling group; though the difference was not significant (*p* > 0.05), likely due to the small sample size and data variations. A similar pattern was noted for hair thickness.

The therapeutic benefits of PRP stem from its rich composition of growth factors, including PDGF, insulin‐like growth factor‐1 (IGF‐1), and VEGF, which play key roles in counteracting AGA‐related changes [24, 25, 26, 27]. Since AGA is associated with factors like reduced angiogenesis, scalp microinflammation, excessive sebum production, and decreased IGF‐1 levels, PRP components may help mitigate these effects, thereby supporting hair growth [24, 28, 29, 30].

Previous research suggests PRP exerts its effects through anti‐inflammatory properties, regulation of scalp oil production, stimulation of follicular activity, and overall enhancement of hair growth [24, 31]. AGA has also been linked to dysfunction in the Wnt/beta‐catenin signaling pathway [32], which PRP has been shown to activate through PDGF stimulation, promoting dermal papilla cell proliferation [33].

Findings from other studies align with our results. Rodriguez et al. demonstrated PRP's efficacy in treating male‐pattern baldness by comparing PRP with saline injections. Their evaluation, conducted at baseline, 15 days, and 3 months posttreatment using TricoScan, showed significant improvements in hair density and growth in the PRP group [34]. Similarly, Kumar Jha et al. assessed PRP microneedling in patients with mild to moderate AGA and observed increased hair follicle numbers, improved hair diameter, and high patient satisfaction after 3 months of treatment [19]. Additionally, Gentile et al. reported that microneedling combined with autologous nonactivated PRP effectively stimulated hair regrowth in AGA patients [35].

Although microneedling has been recognized as a beneficial technique for AGA treatment [36], its superiority over PRP injections remains uncertain. Shruti Gupta observed increased hair density in patients treated with PRP microneedling, but the improvement was not statistically significant [19]. Microneedling may enhance PRP effectiveness by inducing micro‐injuries that improve absorption, stimulate collagen production, and enhance blood circulation, all of which contribute to hair growth [21].

Contrary to most studies, Shapiro et al. investigated PRP's impact on AGA by randomly injecting either PRP or saline into different scalp regions of 35 patients. After three monthly treatment sessions and follow‐up at 3 months, they found no significant difference in hair density between the PRP and control groups [37]. Such discrepancies may result from variations in study design, methodology, sample size, and participant characteristics.

A recent study by Özcan et al. (2022) compared PRP application using Dermapen microneedling with intradermal point‐by‐point injection in male patients with AGA. The trial included 62 male participants with Norwood–Hamilton grades II–V, who received four PRP sessions (the first three at 2‐week intervals and the fourth after 1 month). The results demonstrated significant improvements in hair count, density, terminal hair number, and average hair length in both groups after treatment (all *p* < 0.05). However, the Dermapen microneedling group showed significantly better outcomes in terms of anagen hair count, telogen hair count, and average hair length compared to the injection group (*p* < 0.05). These findings align with our study, where the microneedling group also exhibited greater improvements in hair count, albeit without reaching statistical significance. Incorporating this recent evidence supports the potential superiority of automated microneedling over point‐by‐point PRP injection for enhancing hair regrowth parameters in patients with AGA [38].

Another key aspect of this study was assessing patient satisfaction and pain tolerance. While the microneedling group reported slightly higher satisfaction and better pain tolerance than the PRP injection group, the differences were not statistically significant. Yepuri et al. found that microneedling caused less discomfort than conventional treatments [39], and Nagaratna et al. reported similar findings when PRP was combined with microneedling [40].

Despite certain limitations, such as a relatively small sample size and a short follow‐up duration, this study provides valuable insights into PRP's effectiveness for AGA treatment. By analyzing both individual treatment outcomes and intergroup comparisons, we were able to assess the potential advantages of each method.

## 5 Conclusion

This study supports PRP as an effective treatment for AGA, leading to significant increases in hair count and thickness. Both PRP injection and microneedling approaches yielded positive outcomes, with microneedling showing a greater improvement in hair count; although the difference was not statistically significant. Further large‐scale studies are needed to determine whether microneedling provides superior results.

## Author Contributions

Contributions to the current study include M.R., E.B., A.J., and S.Z. in study idea and design, in the literature review, and drafting and revising the manuscript critically for important intellectual content. M.N. and SO.Z. are involved in drafting the revised manuscript and literature review, and in the analysis and interpretation of the revised version and drafting the manuscript. A.J. is involved in the proposal preparation, statistics, analysis, and drafting the revised manuscript. S.Z. and M.R. are involved in study supervision, data gathering, and literature review. All authors have read and approved the final version to be published and agreed to be accountable for all aspects of the work. All authors agreed on the order in which their names are listed in the revised manuscript.

## Ethics Statement

All collected data were kept confidential and analyzed without the use of specific names. The study adhered to the ethical principles outlined in the Helsinki Declaration. The project was registered at Tehran University of Medical Sciences under registration number IRCT20200127046282N6, with the scientific title “Phase I Clinical Trial: Comparing the Efficacy, Safety, Tolerability, and Satisfaction of Platelet‐Rich Plasma (PRP) Injections and Microneedling for the Treatment of Androgenetic Alopecia.” It was approved by the Research Council under ethics code number IR.TUMS.MEDICINE.REC.1400.031.

## Consent

The authors have nothing to report.

## Conflicts of Interest

The authors declare no conflicts of interest.

## Data Availability

The data that support the findings of this study are available from the corresponding author [S.Z.] upon reasonable request; also, all additional files are included in the manuscript.
